# Construction of a microenvironment immune gene model for predicting the prognosis of endometrial cancer

**DOI:** 10.1186/s12885-021-08935-w

**Published:** 2021-11-11

**Authors:** Yichen Wang, Jingkai Zhang, Yijun Zhou, Zhiguang Li, Dekang Lv, Quentin Liu

**Affiliations:** Institute of Cancer Stem Cell, Dalian Medical University, Dalian, 116044 China

**Keywords:** Endometrial cancer, Immune microenvironment, Prognosis, Nomogram, Immune status

## Abstract

**Background:**

Infiltrating immune and stromal cells are important components of the endometrial cancer (EC) microenvironment, which has a significant effect on the biological behavior of EC, suggesting that unique immune-related genes may be associated with the prognosis of EC. However, the association of immune-related genes with the prognosis of EC has not been elucidated. We attempted to identify immune-related genes with potentially prognostic value in EC using The Cancer Genome Atlas database and the relationship between immune microenvironment and EC.

**Methods:**

We analyzed 578 EC samples from TCGA database and used weighted gene co-expression network analysis to screen out immune-related genes. We constructed a protein–protein interaction network and analyzed it using STRING and Cytoscape. Immune-related genes were analyzed through conjoint Cox regression and random forest algorithm analysis were to identify a multi-gene prediction model and stratify low-risk and high-risk groups of EC patients. Based on these data, we constructed a nomogram prediction model to improve prognosis assessment. Evaluation of Immunological, gene mutations and gene enrichment analysis were applied on these groups to quantify additional differences.

**Results:**

Using conjoint Cox regression and random forest algorithm, we found that TRBC2, TRAC, LPXN, and ARHGAP30 were associated with the prognosis of EC and constructed four gene risk models for overall survival and a consistent nomogram. The time-dependent receiver operating characteristic curve analysis revealed that the area under the curve for 1-, 3-, and 5-y overall survival was 0.687, 0.699, and 0.76, respectively. These results were validated using a validation cohort. Immune-related pathways were mostly enriched in the low-risk group, which had higher levels of immune infiltration and immune status.

**Conclusion:**

Our study provides new insights for novel biomarkers and immunotherapy targets in EC.

**Supplementary Information:**

The online version contains supplementary material available at 10.1186/s12885-021-08935-w.

## Background

Endometrial cancer (EC) is a common gynecological malignant tumor [1]. In recent years, the incidence of EC has increased, with a trend of occurrence in younger generations [2].

**Fig. 1:**
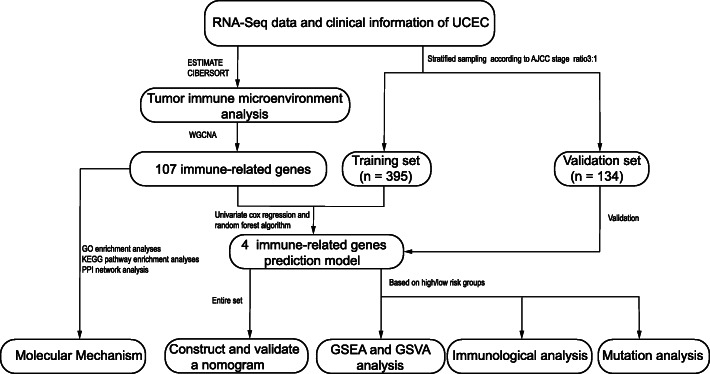
Flowchart of the whole study

The main causes of EC include obesity and endocrine disorders [3, 4]. The immune microenvironment has been reported to significantly affect the biological behavior and prognosis of EC [5–7]. Therefore, the expression of inflammatory genes might be associated with the prognosis of EC. However, prognostic models associated with the expression of immune genes in EC have not yet been established [1]. The establishment of new genome-sequencing technologies and genomic databases has enabled the discovery of tumor biomarkers. Several studies have attempted to evaluate the prognostic value of infiltrating immune and stromal cells in malignancies (hepatocellular carcinoma, renal cell carcinoma and osteosarcoma) [8–10]. Thus, we attempted to identify immune-related genes with potentially prognostic value in EC using The Cancer Genome Atlas (TCGA) database and the relationship between immune microenvironment and EC. This may provide strategies for the development of new immunotherapy modalities for patients with EC. Figure 1 shows an overview of our study, including the steps involved in data preparation, processing, analysis, and validation.

## Methods

### Data sources and preprocessing

TCGA-UCEC-standardized FPKM data (https://bioinformatics.mdanderson.org/), survival data, and clinical information were downloaded from the UCSC Xena official website (https://xena.ucsc.edu/). The TME was assessed in 543 EC samples using the ESTIMATE package in R (version 4.0.2; https://www.r-project.org) [11]. Gene expression datasets were prepared using standard annotation files and uploaded to the CIBERSORT web portal (http://cibersort.stanford.edu/), with the algorithm based on the default signature matrix at 1000 permutations. After converting the gene expression matrix into the immune cell matrix and applying filtering criteria for gene transcription (*P* < 0.05) in CIBERSORT (Perm = 1000), 283 samples (6 normal and 277 tumor tissues) were selected to visualize the matrix of 22 immune cell fractions.

### Visual display of immune cell types

The matrices of 22 immune cell subsets, their correlations, and gene expression profiles were visualized as barplots, boxplots, heat maps, and t-SNE using the ggpubr, corrplot, and Rtsne R packages [12].

### Evaluation of EC-infiltrating immune cells and the TME

ESTIMATE is a tool used for predicting tumor purity and the presence of infiltrating stromal/immune cells in the TME based on gene expression data. The ESTIMATE algorithm is based on single-sample gene set enrichment analysis (ssGSEA), generating three scores, namely, stromal cell scores, immune cell scores, and ESTIMATE scores (which have been shown to exhibit a higher correlation with tumor purity than stromal-only and immune-only scores).

### Screening representative genes in the EC immune microenvironment by weighted gene co-expression network analysis (WGCNA)

We employed the WGCNA to screen for genes with a median absolute deviation of the top 75% and genes with MAD > 0.01 to analyze the immune score-related modules. We then used the WGCNA R package to construct a gene co-expression network, using genetic methods to generate a dynamic shear module, and performed cluster analysis of the module [13]. Genes with similar expression levels were assigned to the same module, with the important parameters being minModuleSize = 30 and mergeCutHeight = 0.25. Our results showed that genes in the four modules (salmon, red, royal blue, and purple) had a high correlation with the immune score (*P* < 0.05). All genes with GS correlation > 0.2 and MM correlation > 0.8 were extracted in the immune-related modules.

### Kyoto Encyclopedia of Genes and Genomes (KEGG) and Gene Ontology (GO) enrichment analyses

Data analysis was performed using clusterProfiler packages in R [14]. A false discovery rate (FDR) of < 0.05 was set as the cut-off criterion indicating significant enrichment of functional GO terms and KEGG pathways.

### Protein–protein interaction (PPI) network and hub gene selection

The cytoHubba (version 0.1) plug-in of Cytoscape (version 3.7.2) was used to screen out hub genes based on degree. We constructed a PPI network using STRING (https://string-db.org/). ClueGO is a plug-in of Cytoscape to visualize nonredundant biological terms for large clusters of genes in a functionally grouped network. We also performed the GO analysis of key genes and visualized the biological processes using ClueGO (version 2.5.4). In addition, we constructed a hierarchical cluster of key genes.

### Selection and verification of prognosis-related genes

The entire cohort (*n* = 529) was divided into the training (*n* = 395) and validation (*n* = 134) groups at a 3:1 ratio based on stratified sampling according to AJCC staging. We used the expression data of immune-related genes transformed by log_2_(FPKM + 1) and the corresponding clinical information to screen out the prognosis-related genes using univariate Cox proportional hazards regression analysis in the training cohort (Hazard Ratio [HR] ≠ 1, FDR < 0.05). Then, prognosis-related genes were used in the subsequent analysis. We applied the random forest algorithm to construct a multigene signature for predicting prognosis in EC using the “randomForest” package of R software [15]. The top four genes with high IncNodePurity value were selected for the downstream analysis. Subsequently, multivariate Cox regression was employed to construct the prognosis-related gene model. Finally, we performed 100 stratified samplings of all patients (*n* = 395). Univariate Cox regression analysis was performed in each group, and prognostic-related genes were sorted according to FDR to verify the prognostic value of the four genes. In each group, the patients were grouped according to their risk scores, and then Kaplan-Meier analysis was performed to verify the prognostic value of the signature through the *P*-value.

### Establishment and validation of the multigene prognostic signature

The training cohort was used for constructing the prognostic risk model, and the validation cohort was used to verify the fit of the model. The risk score was calculated as follows: risk score = h(t, mRNA) = h_0_(t) * exp. (Exp.mRNA_1_ * β_1_ + Exp.mRNA_2_ * β_2_ + … + Exp.mRNA_n_ * β_n_). The h(t, mRNA) is the hazard function determined by a set of covariates (mRNA_1_, mRNA_2_, …, mRNA_n_); The h_0_(t) (always a constant) is the baseline hazard function; The exp.() is exponential function; The Exp.mRNA represents the key gene expression level; the β represents the regression coefficient calculated by multivariate Cox regression. Patients were then stratified into high- and low-risk groups by risk score. We used the Kaplan–Meier survival analysis with log-rank test and tROC analysis to validate the multigene prognostic signature.

### Construction and validation of a gene prognostic nomogram

A composite nomogram was constructed based on all independent prognostic parameters screened using the above multivariate Cox proportional hazards regression analysis to predict the probability of 1-, 3-, and 5-y OS. Then, the predictive accuracy of the nomogram was assessed by employing the timeROC package of R software. We used a bootstrap method with 1000 resamples to generate a calibration curve to visualize the performance of the nomogram with the observed rates of the entire cohort at the corresponding time points.

### Evaluation of immune status

The immune status of each sample was quantified using the single-sample gene-set enrichment analysis (ssGSEA) with 29 immune-related gene signatures (Supplementary Table 1). ssGSEA was conducted using R package “GSVA” [16]. We then analyzed the expression of a pool of key immune checkpoint molecules (CD274, PDCD1, CTLA4, LAG3, HAVCR2, TIGIT, CD27, CD40, CD70, TNFRSF14, CD276, VTCN1, IDO1, PDCD1LG2, CD86, and ICOS) between the low- and high-risk groups.

### Gene set enrichment analysis (GSEA)

As for the ultimate prognosis-related genes used, we performed the GSEA to identify potential biological pathways. The entire cohort of 529 EC samples was divided into two groups based on the risk group analysis. Using the DESeq2 R package [17], we compared the differentially expressed genes (DEGs) between the low- and high-risk groups, and obtained an expression matrix of 8320 genes (FDR < 0.05), sorted by fold change. GSEA (v4.1, http://software.broadinstitute.org/gsea/) was then performed using the JAVA 8.0 platform. The c5.bp.v7.1.symbols.gmt-annotated gene set obtained from the MSigDB was chosen as the reference set to calculate the enrichment score (ES), which estimated whether genes from the previously defined gene sets were enriched in the high−/low-risk groups. The number of permutations was set to 1000. Gene sets with less than 15 genes or more than 500 genes were excluded. A gene set was considered as an enriched group when the normalized *P* value was < 0.05 and FDR was < 0.25.

### Gene set variation analysis (GSVA)

Pathway analyses were performed on the 50 hallmark pathways described in h.all.v7.2.symbols_hallmarks.gmt of the molecular signature database (MSigDB); they were exported using the R package “GSEA”. Next, to assign pathway activity estimates to the samples, we performed the GSVA using the standard settings of the R package “GSVA” [16]. Then, we compared the differences in pathway activity between the two groups. A pathway was considered significant when the normalized, adjusted P value was < 0.05 and the absolute t value was > 3.27.

### Stemness analysis

The stemness signature was exported from the R package “scCancer.” We then defined the stemness score as the Spearman correlation coefficient between stemness signature and sample expression.

### Mutation analysis

The WES data of TNBC patients were obtained from TCGA. The mutation data of low- and high-risk groups were analyzed using the R package “maftools” [18]. Somatic mutation sites were filtered under the following conditions: (i) allele frequency > 5%; (ii) sequencing depth > 9; (iii) reads supporting the alternate allele > 2.

### Statistical analysis

Tumor samples were randomly stratified into two groups using the “sample” function of R software. The heatmap of prognostic genes was plotted using the “pheatmap” R package with zero-mean normalization. Two groups of boxplots were analyzed using Wilcoxon test. Accordingly, Kaplan–Meier curves, *P*-values, and HRs with 95% confidence intervals (CIs) were generated using log-rank tests and univariate Cox proportional hazards regression. All analytical methods above were performed using R software version 4.0.2. All statistical tests were two-sided. Results with *P* < 0.05 were considered statistically significant.

## Results

### Analysis of immune cell subsets of EC using ESTIMATE and CIBERSORT

**Fig. 2:**
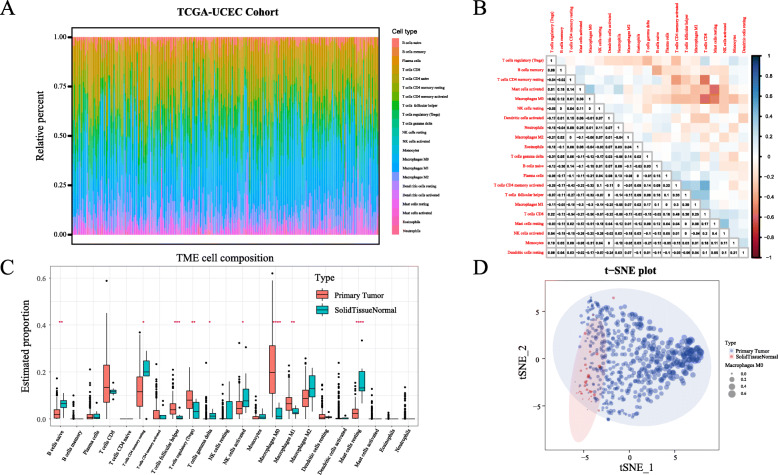
Immune cell subsets in EC analyzed using CIBERSORT. **a** A bar chart displaying the proportion of immune cell subsets. The X-axis shows sample names and the Y-axis shows the percentage of 22 immune cell types, which were predicted separately for each gene expression series. **b** Correlation matrix of 22 immune cell types. **c** Proportion of 22 immune cell types in tumor and normal tissues. Red indicates significant difference. **d** t-SNE analysis performed in all EC cohorts revealed that macrophages (M0) accumulate in higher numbers in tumor tissues than in normal tissues

The analysis of cellular characteristics showed that tumor-related macrophages were the most abundant TME-infiltrating cells, followed by CD8^+^ T-cells. We noted that the numbers of M0 and M1 macrophages were low in normal tissues, but high in cancer tissues (Fig. 2a, c). We also observed that the levels of activated macrophages M0 and CD8^+^ T-cells decreased with EC stage, whereas M1 and M2 macrophages increased with EC stage (Supplementary Fig. 1). The correlation heatmap revealed that CD4^+^ T-cells and M0 macrophages were negatively correlated with resting memory CD8^+^ T-cells, activated mast cells and M0 macrophages were negatively correlated with resting mast cells, and CD8^+^ T-cells were positively correlated with CD4^+^ T-cells (Fig. 2b). Similarly, the obtained boxplot showed an increase in follicular helper T-cells, T-regulatory cells (Tregs), and M0 and M1 macrophages in cancer tissues compared with those in normal tissues. In contrast, we found that naive B-cells, CD4 memory resting T-cells, gamma delta T-cells, activated NK cells, and mast cells showed high abundance in normal tissues, but low abundance in tumor tissues (Fig. 2c). The tSNE plot of 22 immune signatures showed an increased number of M0 macrophages in tumor tissues compared with that in normal tissues (Fig. 2d).

### Construction of weighted co-expression network and identification of gene modules

**Fig. 3:**
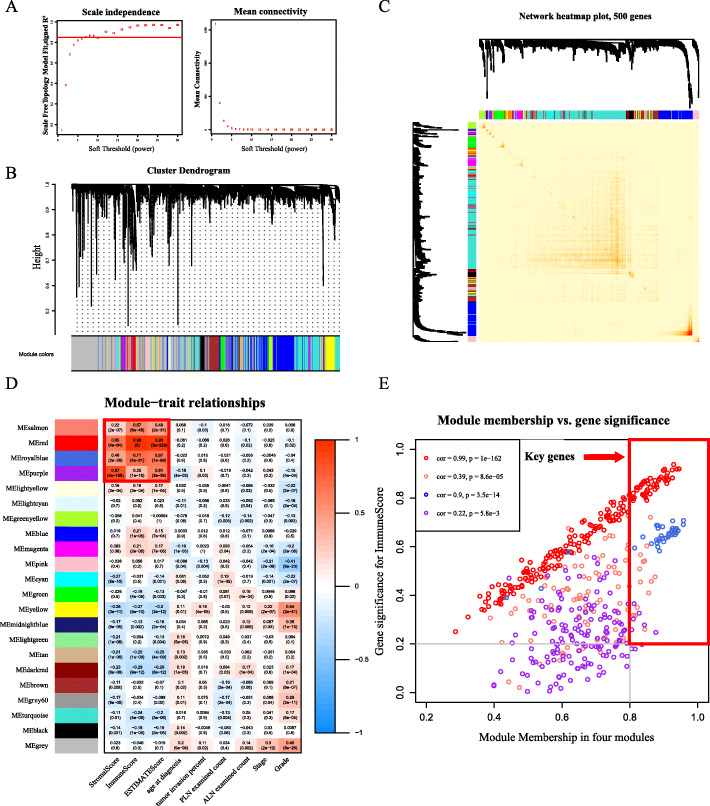
Immune genes (107) were screened out through the analysis of the co-expression network. **a** Analysis of network topology met the scale-free topology threshold of 0.85 when β = 7. **b** Cluster dendrogram and co-expression network modules produced by average linkage hierarchical clustering of mRNAs based on topological overlaps. **c** Heatmap depicts the topological overlap matrix among 500 randomly selected genes in the analysis. **d** Heatmap of the correlation between module genes (rows) and traits (columns). **e** Scatter plot shows the correlation between gene significance (GS) and module membership (MM) in four modules (salmon, red, royal blue, and purple)

We chose a threshold value of 7 for the WGCNA, which had the lowest power of the scale-free topological fit index of 0.85 (Fig. 3a). After merging similar clusters, we identified 21 modules that contained groups of genes with similar patterns of connection strengths with other genes (Fig. 3b, c). Finally, we determined the correlation between these modules and traits (Fig. 3d). A significant association was found between the salmon, red, royal blue, and purple modules and stromal cell, immune cell, and ESTIMATE scores. Evaluating the correlation between GS and MM is key in measuring the quality of the construction of gene modules. After correlating the modules with the ImmuneScore, the correlation between GS and MM in the four modules was observed to reach 0.39, 0.99, 0.9, and 0.22 (Fig. 3e). Thus, we set more stringent screening conditions, namely GS correlation > 0.2 and MM correlation > 0.8, and finally selected 107 key genes for the downstream analyses.

### GO and KEGG functional enrichment analyses

Four hundred and thirty-four GO terms and 37 KEGG pathways were indicated in 107 key genes. The KEGG pathway analysis showed that the top 15 significantly enriched pathways were related to hematopoietic cell lineage, phagosome pathways, and the human T-cell leukemia virus 1 infection pathway (Supplementary Fig. 2a). The top five GO terms of each subclass were found to be mainly related to immune activation, such as T-cell activation and proliferation (Supplementary Fig. 2b).

### PPI network of key genes

To better understand the interplay among the key genes, we constructed a PPI network, which revealed that all key genes were densely interconnected. The PPI network of the top 20 key genes sorted by degree revealed several gene interactions (Supplementary Fig. 3a). The biological process analysis of the key genes is shown in Supplementary Fig. 3b.

### Establishment of a prognostic risk model of the four immune-related genes

**Fig. 4:**
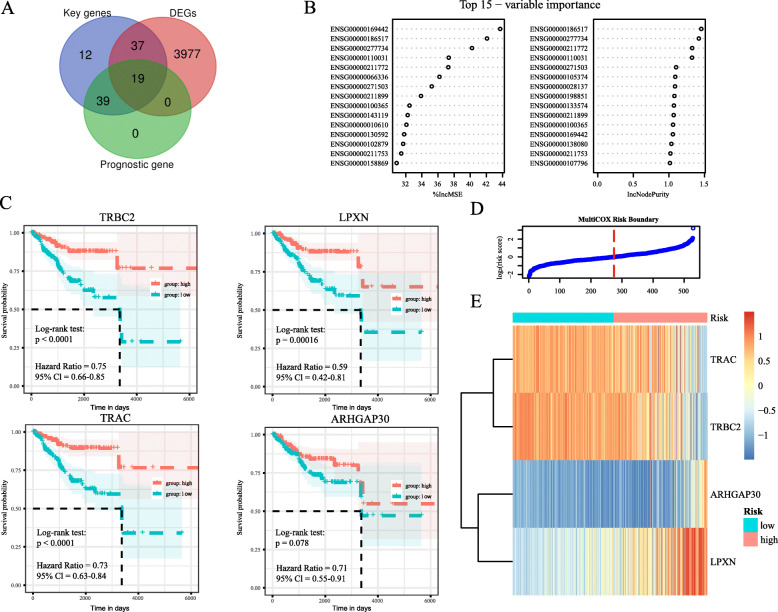
Identification of four immune-related prognostic genes in EC. **a** Venn diagram analysis of key genes based on DEGs and prognostic genes. **b** Random forest algorithm was preformed to further select microenvironment-related prognostic genes. **c** Kaplan–Meier survival analysis of TRBC2, LPXN, TRAC, and ARHGAP30. **d**, **e** The curve of risk score (log2) and expression heatmap of the four immune-related genes in the high- and low-risk groups

The univariate Cox regression analysis of the training group showed that 58 immune-related genes were found to be associated with the OS of patients (*P* < 0.05; Fig. 4a). To verify the reliability of the model, we selected the top four immune-related genes with the highest IncNodepurity using the random forest algorithm (Fig. 4b), with its coefficient in multivariate Cox regression as follows: -0.08485 (LPXN), − 0.18955 (TRBC2), − 0.14632 (TRAC), and 0.13717 (ARHGAP30). We noted significant differences between the high- and low-risk groups. Additionally, up on optimization of the expression median for analysis of the four immune-related genes based on the selected cut-off, we found that the low expression of TRBC2, LPXN, TRAC, and ARHGAP30 was associated a with poor prognosis (Fig. 4c). We then calculated the risk score for each patient, with 1 as the cut-off point, and divided the patients into the high-risk (risk score > 1, *n* = 255) and low-risk (*n* = 274) groups (Fig. 4d); the expression heatmap of these four genes in the two groups is shown in Fig. 4e. Finally, 100 stratified samplings (*n* = 395) were performed on all patients to verify the prognostic performance of the four genes and the model. The results show that the performance of the four genes and the model is good (Supplementary Fig. 4).

### Prognostic analysis of the four immune-related genes classifier and construction of the nomogram prognostic model

**Fig. 5:**
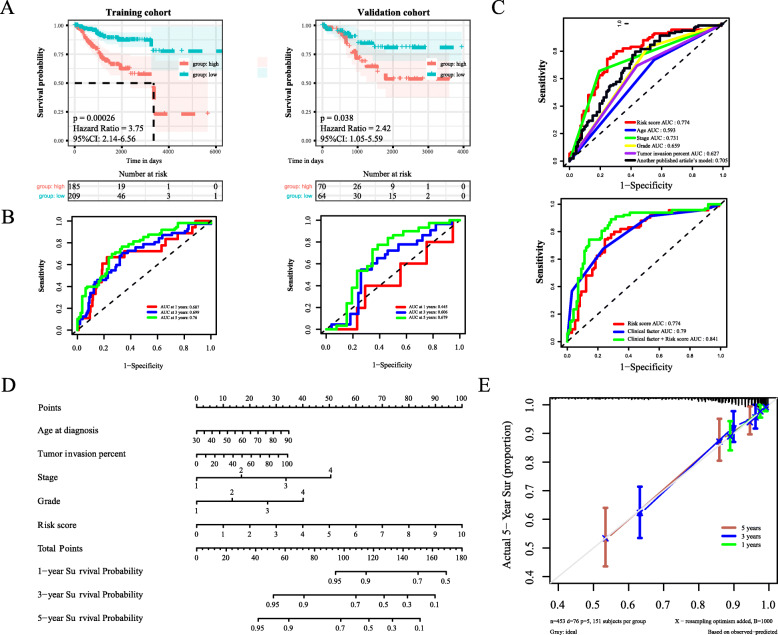
Prognostic analysis of a four immune-related genes classifier and construction of the nomogram prognostic model. **a** Kaplan–Meier analysis of OS according to the four immune-related gene classifier in the training and validation cohorts. **b** Time-dependent ROC analysis of the four immune-related gene classifier for 1-, 3-, and 5-y OS in the training and validation cohorts. **c** Time-dependent ROC analysis for evaluating the prognostic value of risk score and clinical factors (age, stage, grade, and tumor invasion percent). **d** Nomogram integrating the four gene-based risk score and significant clinical traits. **e** Calibration plot of actual risk probability

The Kaplan–Meier analysis of the OS showed that the high-risk group in the training cohort was associated with poor OS (*P* = 0.0026), whereas the OS rate of high-risk patients in the validation cohort was significantly lower than that of low-risk patients (*P* = 0.038) (Fig. 5a). As shown in Fig. 5b, time-dependent receiver operating characteristic (ROC) analysis revealed that the area under the curve (AUC) for 1-, 3-, and 5-y OS in the training cohort was 0.687, 0.699, and 0.76, respectively. The predictive value of the four immune-related gene classifiers was confirmed in the validation cohort, which showed that the AUCs for 1-, 3-, and 5-y OS in the validation cohort were 0.445, 0.606, and 0.679, respectively. To establish a more reliable predictive method for clinical practice, we combined the two cohorts (*n* = 453) and constructed a compound nomogram. We performed univariate and multivariate Cox proportional hazards regression analyses on the relationship between clinical characteristic variables and OS (Supplementary Table 2). Meanwhile, by drawing the ROC curve, we found that risk score has a better predictive ability than other clinical factors for 5-y OS. The AUC of ROC increased after combining the risk score with other clinical factors (Fig. 5c), which suggested that the risk score may be an independent risk factor for patients. We used the 10 genes identified in another published article to build a model for the entire set [19]. The 5-year AUC value of the 10 genes model is lower than that of our model. As shown in Fig. 5d, we employed these variables to analyze the survival probability of patients at 1, 3, and 5 y. To verify the predictive value of the nomogram, we used C-statistics to analyze the generated nomogram model. The C-index of the nomogram was 0.818 (95% CI, 0.865–0.77). In addition, the calibration plot generated for patients with a 1-, 3-, and 5-y OS prediction demonstrated that the predicted outcome of the nomogram showed a good agreement with the actual outcome (Fig. 5e). Finally, we used the online website GEPIA2 to analyze the overall prognosis of four immune-related genes in endemic cancer (Supplementary Fig. 5).

### Tumor microenvironment and immune status

**Fig. 6:**
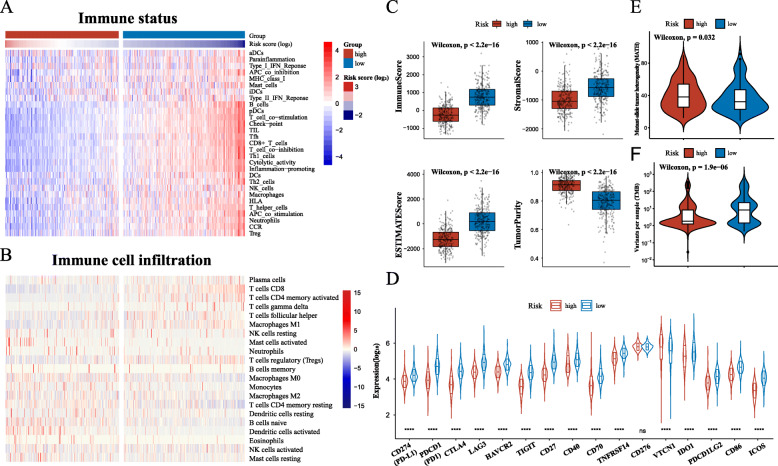
Evaluation of the TME and immune status between the low- and high-risk groups. Heatmap of **a** immune status and **b** immune cell infiltration. **c** Box plot of immune score, stromal score, ESTIMATE score, and tumor purity in the risk groups. **d** The violin plot of immune checkpoints expression in the high- and low-risk groups. Violin plot shows **e** MATH and **f** TMB in the high- and low-risk groups

To understand the immunological evaluation value of the model, we performed immunological analysis between the high- and low- risk groups. The ssGSEA analysis of the immune status of patients showed that type I IFN response was not significantly different between the groups. However, the scores of other immune cells, immune function, and immune pathway gene set were significantly higher in the low-risk group than in the high-risk group, indicating that the immune status of the low-risk group was higher than that of the high-risk group (Fig. 6a, Supplementary Fig. 6a). In addition, we compare the immune infiltration results of CIBERSORT analysis between the groups (Fig. 6b). The results show that the infiltration of T cells CD8, T cells follicular helper, T cells regulatory (Tregs), T cells gamma delta, and macrophages M1 in the low-risk group was significantly higher than that in the high-risk group (Supplementary Fig. 6b). The results of the ESTIMATE analysis showed that the immune score, stromal score, and ESTIMATE score of the low-risk group were significantly higher than those of the high-risk group. Furthermore, the tumor purity was significantly lower than that of the high-risk group, indicating that the degree of immune infiltration in the low-risk group was higher than that in the high-risk group (Fig. 6c). The checkpoint score based on the ssGSEA showed that the low-risk group was more suitable for immunotherapy than the high-risk group. We analyzed the clinical common immune checkpoint expression, tumor mutation burden (TMB), and mutant-allele tumor heterogeneity (MATH) of patients in the two groups, respectively, as references for the evaluation of clinical immunotherapy. The expression of immune checkpoints except CD276, VTCN1, and TMB were higher in the low-risk group than in the high-risk group, and MATH was significantly lower in the low-risk group (Fig. 6d, e, and f). These results indicated that the gene model has a good ability for immune evaluation, and the low-risk group has higher immune infiltration and immune status, which may result in better efficacy of immunotherapy.

### Enrichment analysis, stemness analysis, and mutation profile

**Fig. 7:**
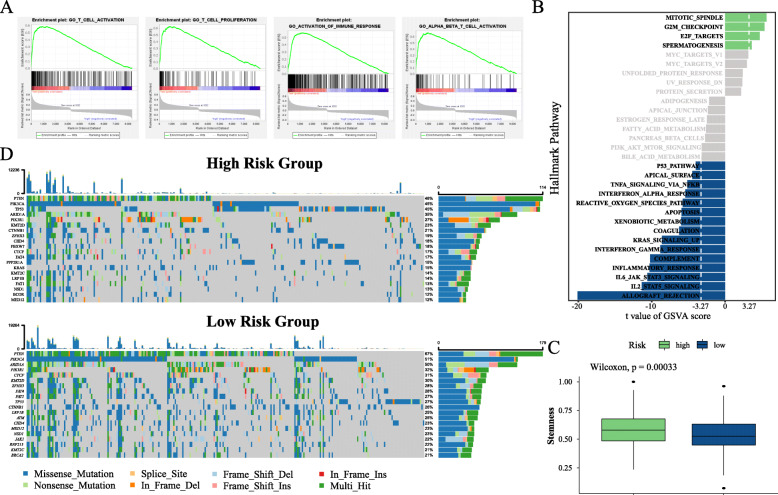
Enrichment, stemness, and mutation analyses. **a** GSEA associated with the risk group. **b** Differences in hallmark pathway activities scored per sample by GSVA between the high- and low-risk groups. **c** Distribution of stemness in the high- and low-risk groups. **d** Mutations of top 20 genes in the high- and low-risk groups

We performed the GSEA to identify potential biological processes in the four immune-related prognostic gene model. Our results revealed that the pathways of T-cell activation, T-cell proliferation, activation of immune response, and alpha beta T-cell activation were enriched in low-risk patients. The details are shown in Fig. 7a. We also generated a heatmap of the transcriptional expression profiles of the 100 DEGs (Supplementary Fig. 7). In addition, Supplementary Table 3 shows the enrichment score and statistical significance of each pathway. The hallmark pathway enrichment results showed that the top six hallmark pathways in the low-risk group were all immune-related (Fig. 7b).

Stemness is one of the factors that determine the malignancy of a tumor. In the analysis of stemness, we found that the low-risk group had lower stemness than the high-risk group (Fig. 7c). This might be one of the reasons for the better prognosis in the low-risk group. In addition, we compared the distribution of histotype between two groups, and the results showed that there were significantly more type II patients in the high-risk group than in the low-risk group (Chi-square test: *p*-value = 9.194e-05; Fisher exact test: *p*-value = 6.936e-05). This may also be one of the reasons for the poor prognosis of the high-risk group. For further research on the potential mechanism of EC in the different risk groups, we analyzed the somatic mutation data of the two groups of patients and showed the mutation profile (Fig. 7d). There were some differences in the top 20 genes between the groups. The top 10 genes of mutation frequency in the high-risk group were PTEN (46%), PIK3CA (45%), TP53 (45%), ARID1A (35%), PIK3R1 (27%), KMT2D (23%), CTNNB1 (21%), ZFHX3 (19%), CHD4 (18%), and FBXW7 (18%). The top 10 genes of mutation frequency in the low-risk group were PTEN (67%), PIK3CA (51%), ARID1A (50%), PIK3R1 (32%), CTCF (31%), KMT2D (30%), ZFHX3 (28%), FAT4 (28%), FAT1 (27%), TP53 (27%), CTNNB1 (26%), and LRP1B (25%). The Fisher exact test on the gene mutation frequency of the two groups revealed that there were differences among these 20 genes (adjusted *P* < 0.05) and that TP53 had a high mutation frequency in the high-risk group. On the contrary, some genes had a high mutation frequency in the low-risk group, such as PTEN, FAT1, CTCF, ARID1A, and LRP1B. These results indicate that our gene model has good performance and could be considered as a candidate factor for clinical indicators.

## Discussion

The incidence of EC has been increasing annually, ranking it the first among gynecological malignancies in developed nations [20, 21]. The treatment options for EC include surgery, radiotherapy and chemotherapy, hormone therapy, and targeted therapy [22, 23]. However, treatment efficacy has been reported to be reduced in patients with recurrent or specific pathological types of EC [24]. Abundant infiltrating immune cells and cytokines, which can stimulate endogenous antitumor immune responses, have been typically observed in EC tissues, indicating that the immune microenvironment can influence the prognostic survival of patients with EC [25].

In this study, we performed TCGA data mining to reveal correlations between the infiltration pattern of immune cells into the TME and the clinical characteristics of patients with EC. Our results showed that a large number of immune cells infiltrated the interstitium of EC, among which M0 macrophages, followed by CD8^+^ T-cells, were found to be the most common immune cells. These cells are known to be important regulators of the TME, playing an important role in the occurrence, progression, and prognosis of EC. Interestingly, the proportion of M0 macrophages in tumor and normal tissues was significantly different (*P* < 0.001). However, as the tumor progressed, the proportion of M0 macrophages gradually decreased, whereas that of M2 macrophages gradually increased, indicating that TME signals emitted by tumor or mesenchymal cells can polarize undifferentiated macrophages into M2 macrophages (tumor-associated macrophages, TAMs) [26, 27]. In particular, TAMs are known to pass through multiple signals and promote the occurrence and development of tumors, which is an important factor implicated in the poor prognosis of EC [28].

The PPI analysis revealed that the top 20 hub genes in the EC microenvironment were related to integrin, immune signaling adapter, and leukocyte surface antigen pathways. We found that 16 of the 20 hub genes were associated with patient survival (*P* < 0.05). TYROBP (transmembrane immune signaling adapter TYROBP), which was the second highest interconnected node in the PPI network revealed to be negatively associated with OS, was found to be upregulated in EC tumor tissues. TYROBP is known to be tyrosine-phosphorylated in the ITAM domain following ligand binding by the associated receptors, leading to the activation of additional tyrosine kinases and subsequent cell activation [29]. The intersected genes screened out among key genes and prognosis-related genes in the training cohort using univariate Cox regression methods were used for random forest algorithm to identify the four genes (*TRAC*, *TRBC2*, *LPXN*, and *ARHGAP30*) significantly associated with the OS of patients with EC. Both *TRAC* and *TRBC2* have been reported to express the alpha and beta chains of the T-cell receptor [30–33]. The alpha and beta T-cell receptors are specific antigen receptors that are essential for the immune response and are present on the surface of T-lymphocytes for the recognition of peptide-major histocompatibility complexes (MHCs) displayed by antigen-presenting cells (APCs). In addition, we analyzed these four genes through online websites to explore the performance of these genes in other cancers. These genes have been found to have good prognostic evaluation capabilities in endemic cancer, such as head and neck squamous cell carcinoma, skin cutaneous melanoma, thymoma, breast invasive carcinoma, etc. This also shows that our screening results are reliable. After identifying these four prognostic genes, we developed a four-gene prognostic signature and investigated its prognostic value in patients with EC. In the application of the model, patients with a risk score great than 1 were considered to be at high risk. Patients in the high-risk group showed a significantly worse prognosis than those in the low-risk group. The AUC value of the prognostic model (AUC = 0.774) was greater than that of stage (AUC = 0.731), grade (AUC = 0.659), tumor invasion percent (AUC = 0.627) and age (AUC = 0.593). This indicates that the prognostic model is more accurate than the commonly used clinical prognostic indicators. We then built a nomogram incorporating the risk score, tumor invasion percentage, age, AJCC stage, and grade to visualize the prognosis of patients with EC. This can be used to predict the individual 1-, 3-, and 5-y OS probability specifically according to the risk score and other conventional clinical prognostic parameters; therefore, our prognostic model may help clinicians decide on better EC treatments.

TME plays an important role in tumor development [34, 35]. Therefore, the study of the molecular composition and function of tumor microenvironment is of great importance for the assessment of the progression and immune response of EC. Immune score and stromal score were obtained through the ESTIMATE analysis, and the ESTIMATE score and tumor purity were predicted based on these two scores. The results showed that immune cell infiltration was higher in the low-risk group than in the high-risk group. The results of the CIBERSORT analysis indicate that the infiltration of T cells CD8, T cells follicular helper, T cells regulatory (Tregs), T cells gamma delta, and macrophages M1 in the low-risk group was significantly higher than that in the high-risk group. Through the ssGSEA, we analyzed the enrichment of genes of immune cells, immune function, and immune pathway activity in each sample. The results of the ssGSEA revealed that the immune activity of the low-risk groups was higher than that of the high-risk group. Moreover, the GSEA and hallmark pathway analysis showed that immune-related pathways, including the activation of the immune response and molecular signals induced by it were enriched in the low-risk group. These results indicate that the immune ability of patients was stronger in the low-risk group than in the high-risk group, indicating a better prognosis. Hence, by exploring the response of the immune checkpoint inhibitors, we found that the expression of 14 immune checkpoints and TMB were significantly higher in the low-risk group than in the high-risk group, and the MATH of the low-risk group was lower than that of the high-risk group. Several recent studies have demonstrated that TMB can be used as a biomarker to predict patient response to immune checkpoint inhibitors. Tumor heterogeneity is also closely related to tumors, and compared with factors such as TMB, tumor heterogeneity has a greater impact on the effect of immunotherapy [36, 37]. According to these findings, we speculate that EC patients with a low-risk score might have a better response to these checkpoint inhibitors.

Nevertheless, there were some limitations associated with our study. First, the present study was completely based on data obtained from TCGA database. Thus, the results should be validated using external databases and additional clinical-based experiments. Second, the predictive value of the immune-related prognostic model should be experimentally tested using a large number of EC samples. Third, our study only focused on large-scale mRNA sequencing data from TCGA platform. However, other types of data, such as copy number variations, and DNA methylation, are also provided in the public dataset. If possible, these four novel biomarkers should be further analyzed to determine whether their expression levels are associated with the mutation types mentioned above.

## Conclusions

In the present study, by integrating transcriptome and clinical data, we identified a novel prognostic model for EC. Combined with immunological and genomic analysis, we demonstrate that this novel immune gene model could potentially be used in clinical practice for assessing individual risk of death and potentially guide treatment strategies.

## Supplementary Information

**Additional file 1.**

**Additional file 2.**

**Additional file 3.**

**Additional file 4.**

**Additional file 5.**

**Additional file 6.**

**Additional file 7.**

**Additional file 8.**

**Additional file 9.**

**Additional file 10.**

## Data Availability

The TCGA-UCEC-standardized FPKM data used during the current study are available at: https://bioinformatics.mdanderson.org/. WES data obtained from the TCGA database is available at: https://portal.gdc.cancer.gov/projects. Survival data and clinical information are available from the UCSC Xena official website (https://xena.ucsc.edu/).

## Abbreviations

ARHGAP30: Rho GTPase activating protein 30
EC: Endometrial cancer
ESTIMATE: Estimation of STromal and Immune cells in MAlignant Tumors using Expression data
GO: Gene Ontology
LPXN: Leupaxin, paxillin protein family
KM: Kaplan–Meier
MATH: Mutant-allele tumor heterogeneity OS overall survival
ROC: Receiver operating characteristic
TCGA: The Cancer Genome Atlas
TMB: Tumor mutation burden
TME: Tumor microenvironment
TRAC: T-cell receptor alpha constant
TRBC2: T-cell receptor beta constant 2
WES: Whole exome sequencing
WGCNA: Weighted gene co-expression network analysis.
