# Supplementary material for: Construction of a microenvironment immune gene model for predicting the prognosis of endometrial cancer
Source: BMC Cancer. 2021 Nov 11;21:1203. doi: 10.1186/s12885-021-08935-w (PMC8588713; doi:10.1186/s12885-021-08935-w)
Supplement: Supplementary file 4 — Additional file 4. [file 12885_2021_8935_MOESM4_ESM.pdf]

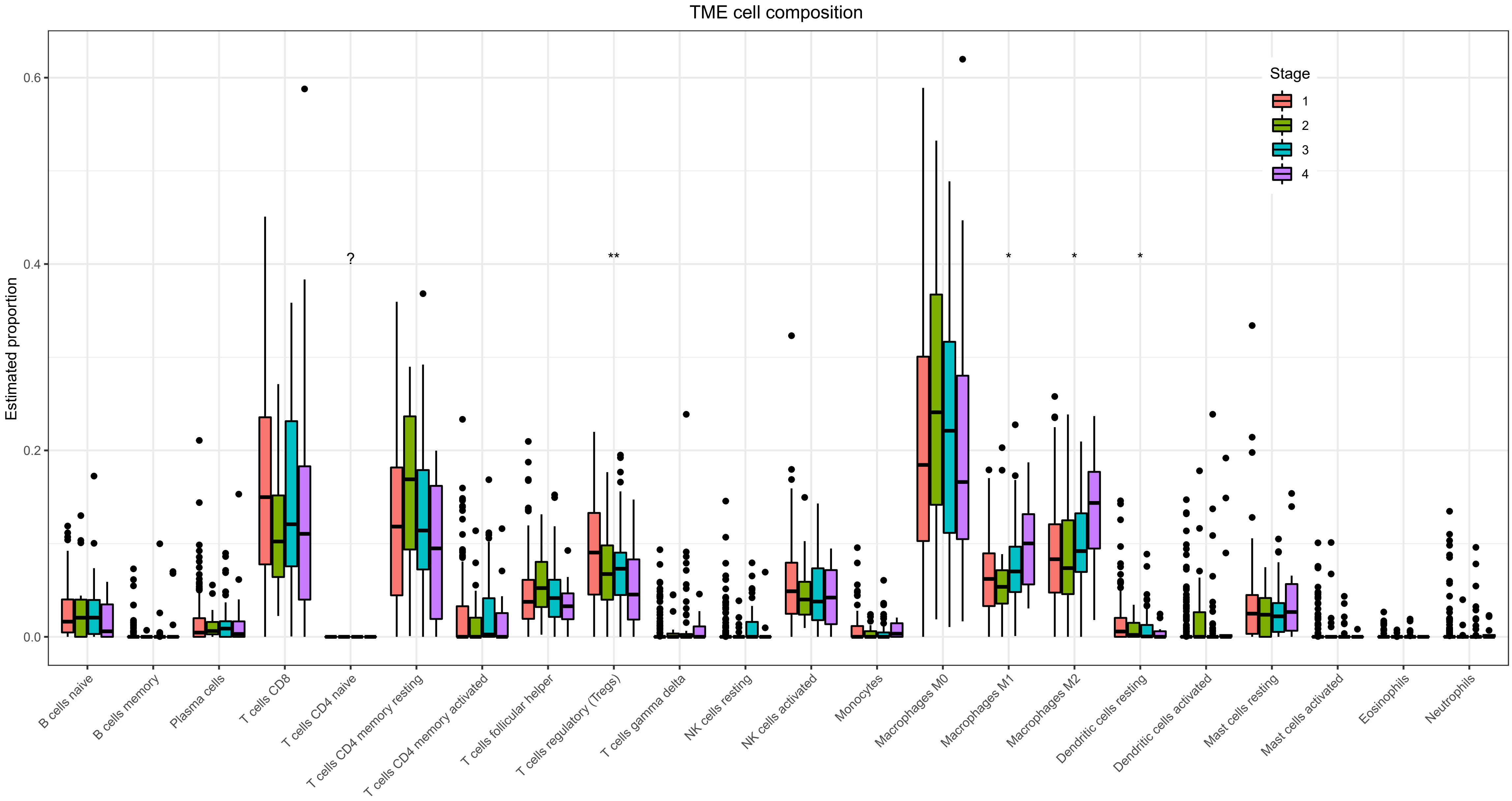

Supplementary Figure 1. Proportions of 22 immune cell types in different tumor stage.
