# Supplementary material for: Construction of a microenvironment immune gene model for predicting the prognosis of endometrial cancer
Source: BMC Cancer. 2021 Nov 11;21:1203. doi: 10.1186/s12885-021-08935-w (PMC8588713; doi:10.1186/s12885-021-08935-w)
Supplement: Supplementary file 5 — Additional file 5. [file 12885_2021_8935_MOESM5_ESM.pdf]

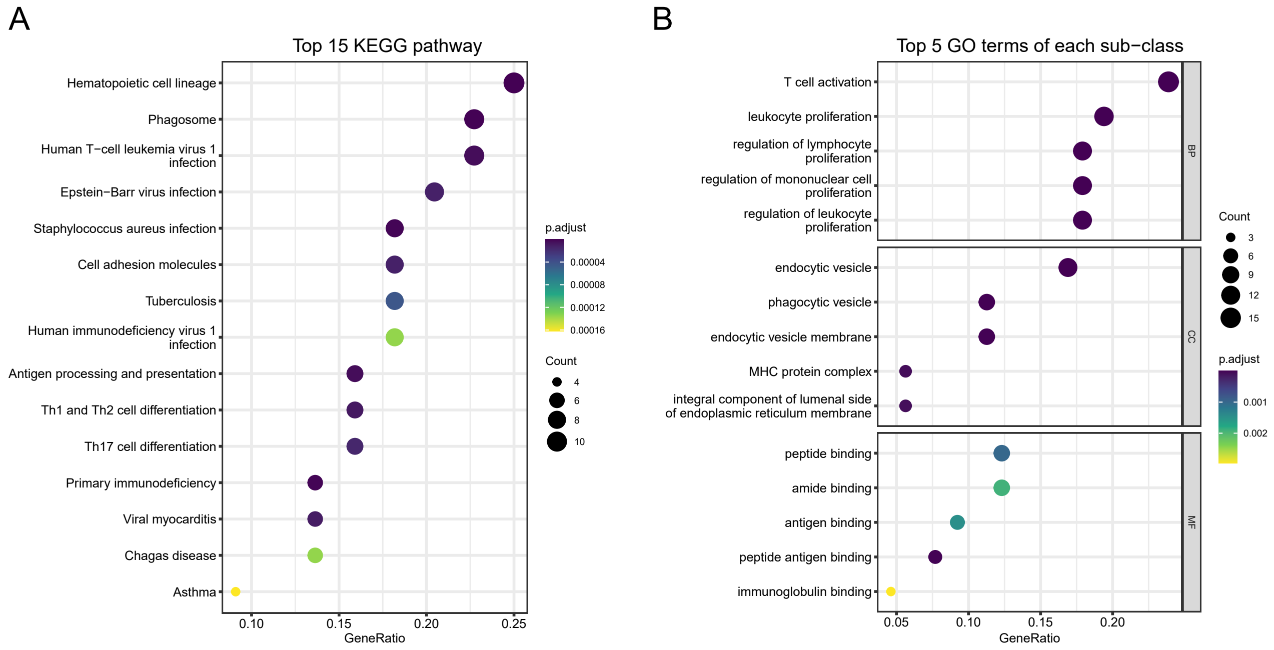

Supplementary Figure 2. Functional enrichment analysis of 107 key genes. (a) The 15 KEGG pathway of key genes. (b) The top 5 GO terms of each sub-class.
