# Supplementary material for: Construction of a microenvironment immune gene model for predicting the prognosis of endometrial cancer
Source: BMC Cancer. 2021 Nov 11;21:1203. doi: 10.1186/s12885-021-08935-w (PMC8588713; doi:10.1186/s12885-021-08935-w)
Supplement: Supplementary file 6 — Additional file 6. [file 12885_2021_8935_MOESM6_ESM.pdf]

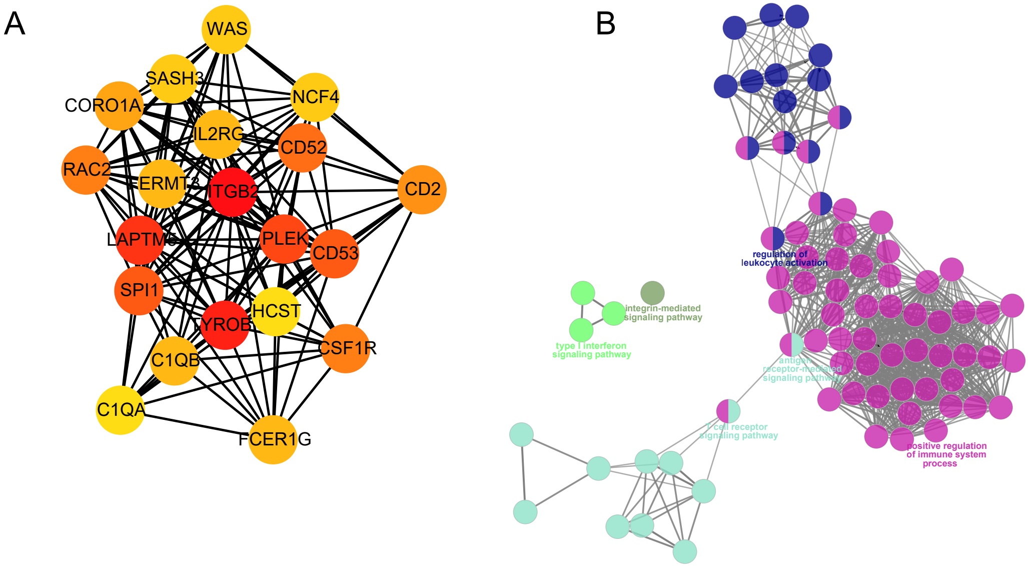

Supplementary Figure 3. Interaction network and analysis of the key genes. (a) Screen out the 20 most important hub genes using the cytoscape software plugin cytoHubba. (b) The biologic process functional annotation analysis of key genes was performed by ClueGO. Different colors of nodes refer to the functional annotation of ontologies. Corrected P value < 0.05 was considered statistically significant.
