# Supplementary material for: Construction of a microenvironment immune gene model for predicting the prognosis of endometrial cancer
Source: BMC Cancer. 2021 Nov 11;21:1203. doi: 10.1186/s12885-021-08935-w (PMC8588713; doi:10.1186/s12885-021-08935-w)
Supplement: Supplementary file 7 — Additional file 7. [file 12885_2021_8935_MOESM7_ESM.pdf]

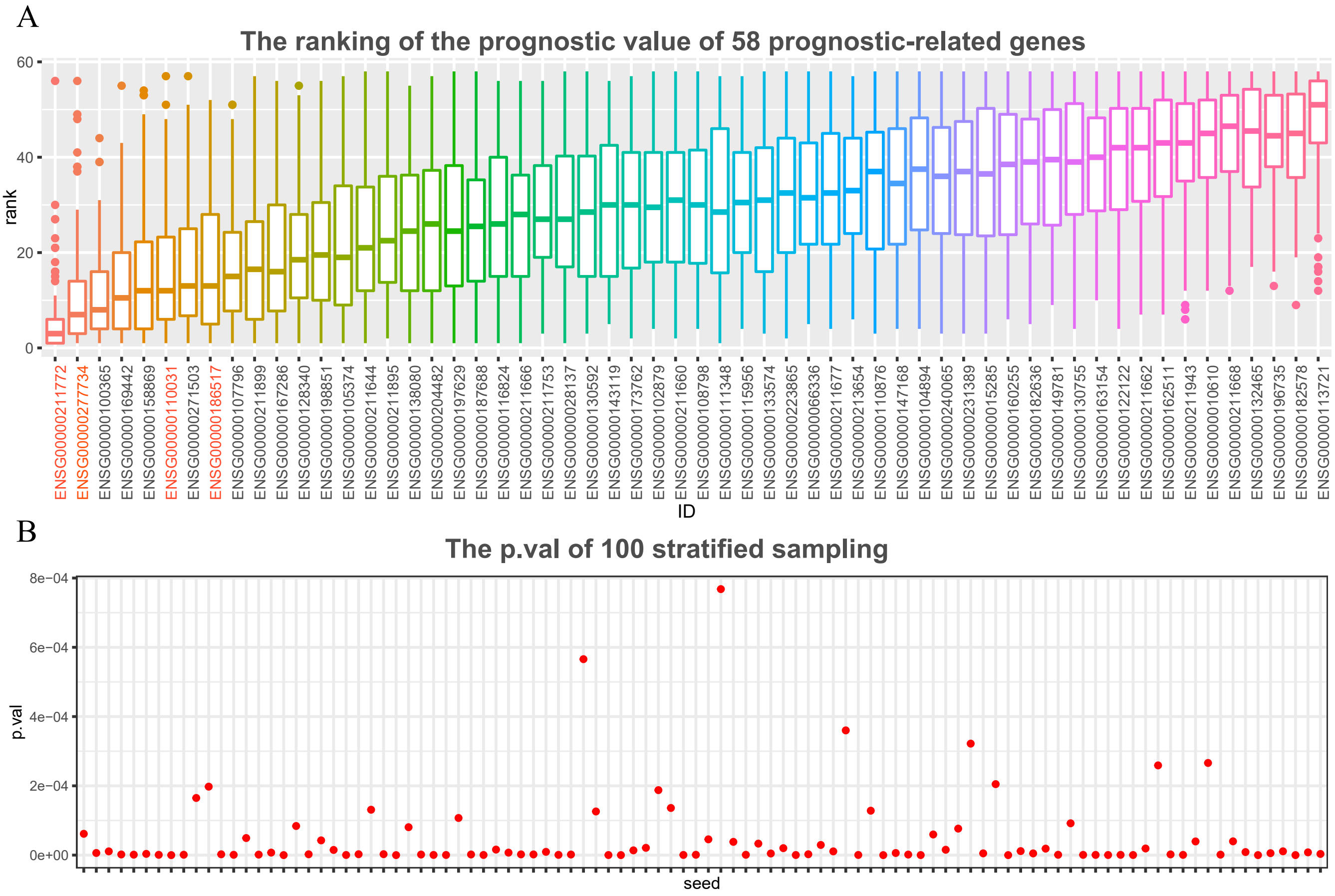

Supplementary Figure 4. Stratified sampling verification. (a) Box plot of 58 prognostic-related genes ranking. (b) Scatter plot of the p value distribution of the model after 100 stratified sampling .
