# Supplementary figures and images for: Construction of a microenvironment immune gene model for predicting the prognosis of endometrial cancer
Source: BMC Cancer. 2021 Nov 11;21:1203. doi: 10.1186/s12885-021-08935-w (PMC8588713; doi:10.1186/s12885-021-08935-w)

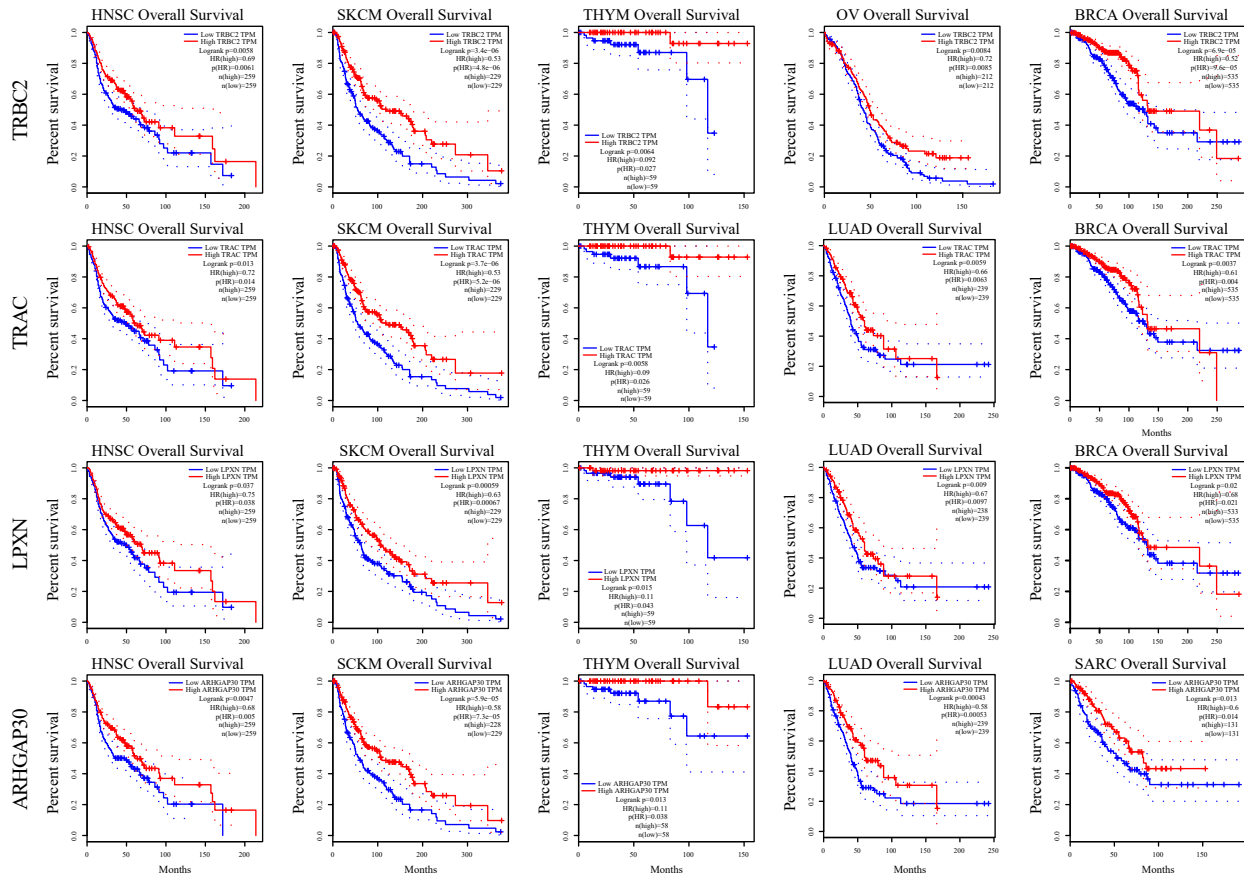

Supplementary Figure 5. Survival analysis of four immune-related genes in other types of cancer.

Supplement: Supplementary file 8 — Additional file 8. [file 12885_2021_8935_MOESM8_ESM.pdf]
