# Supplementary material for: Construction of a microenvironment immune gene model for predicting the prognosis of endometrial cancer
Source: BMC Cancer. 2021 Nov 11;21:1203. doi: 10.1186/s12885-021-08935-w (PMC8588713; doi:10.1186/s12885-021-08935-w)
Supplement: Supplementary file 9 — Additional file 9. [file 12885_2021_8935_MOESM9_ESM.pdf]

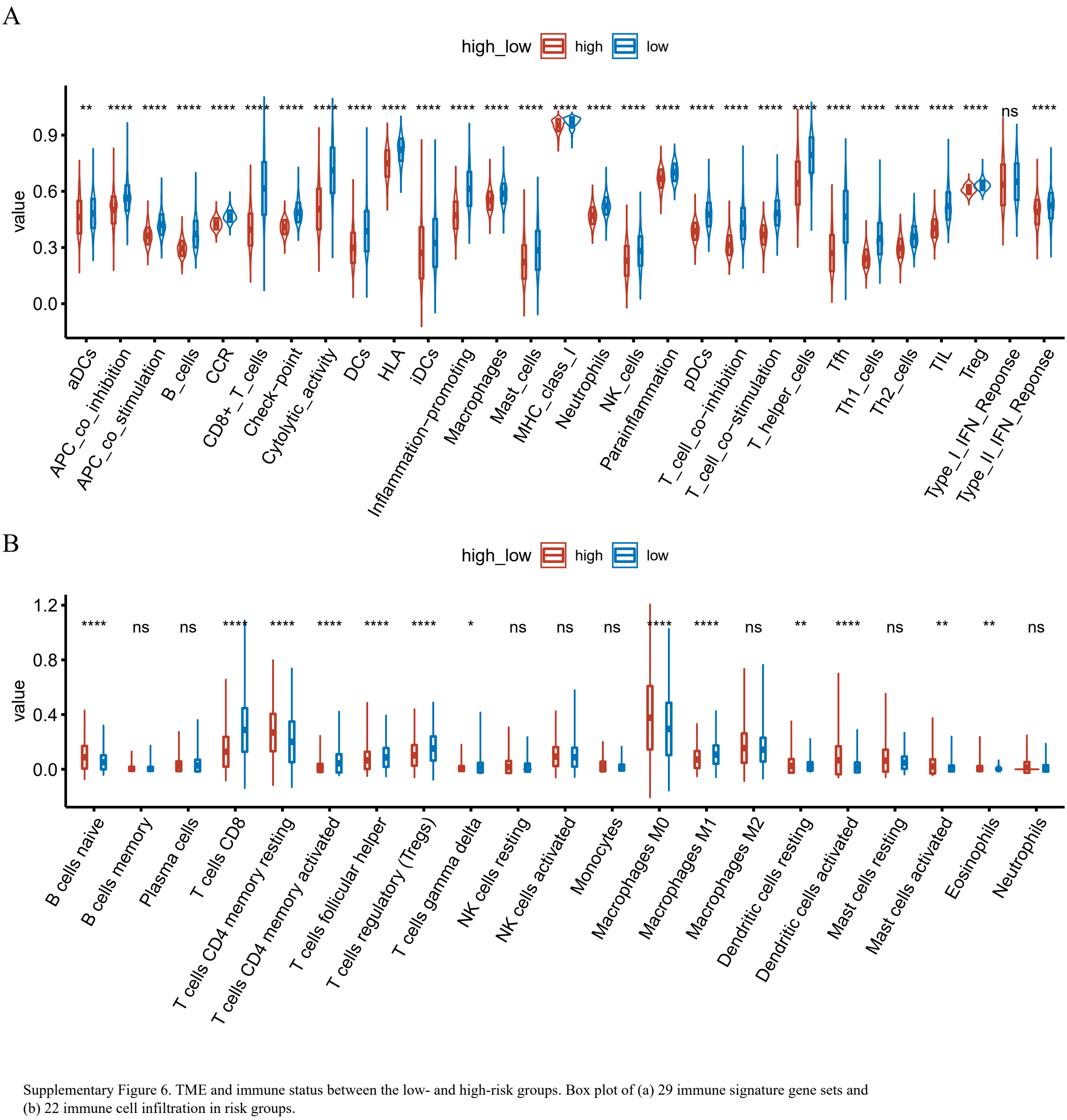

Supplementary Figure 6. TME and immune status between the low- and high-risk groups. Box plot of (a) 29 immune signature gene sets and (b) 22 immune cell infiltration in risk groups.
