# Supplementary material for: Construction of a microenvironment immune gene model for predicting the prognosis of endometrial cancer
Source: BMC Cancer. 2021 Nov 11;21:1203. doi: 10.1186/s12885-021-08935-w (PMC8588713; doi:10.1186/s12885-021-08935-w)
Supplement: Supplementary file 10 — Additional file 10. [file 12885_2021_8935_MOESM10_ESM.pdf]

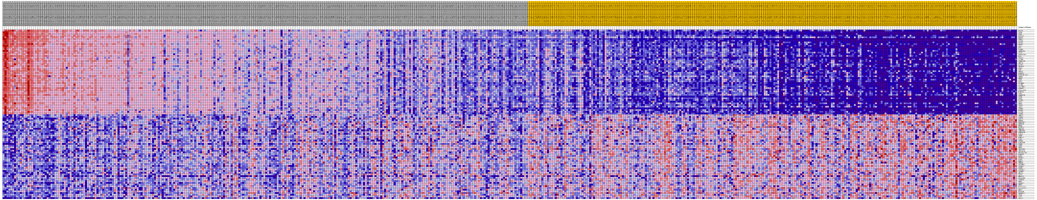

Supplementary Figure 7. Transcriptional expression profiles of the 100 significant genes were performed in a heat map.
